# 3D *In Vitro* Models for Investigating the Role of Stiffness in Cancer Invasion

**DOI:** 10.1021/acsbiomaterials.0c01530

**Published:** 2021-06-03

**Authors:** Auxtine Micalet, Emad Moeendarbary, Umber Cheema

**Affiliations:** † Department of Mechanical Engineering, University College London (UCL), Torrington Place, London, U.K. WC1E 6BT; ‡ Department of Biological Engineering, Massachusetts Institute of Technology (MIT), Cambridge, Massachusetts 02139, United States; § Division of Surgery and Interventional Sciences, UCL Centre for 3D Models of Health and Disease, University College London (UCL), Charles Bell House, London, U.K. W1W 7TS

**Keywords:** stiffness, cancer, invasion, tissue engineering, biomechanics, tumour microenvironment

## Abstract

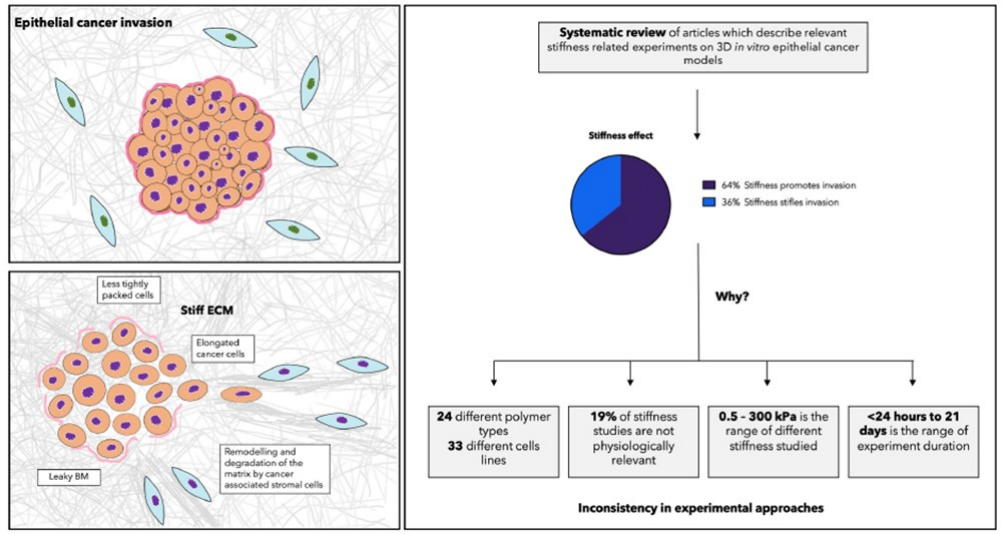

Background: Tumorigenesis is attributed to the interactions of cancer cells with the tumor microenvironment through both biochemical cues and physical stimuli. Increased matrix deposition and realignment of the collagen fibers are detected by cancer cells, inducing epithelial-to-mesenchymal transition, which in turn stimulates cell motility and invasiveness. Methods: This review provides an overview of current research on the role of the physical microenvironment in cancer invasion. This was achieved by using a systematic approach and providing meta-analyses. Particular focus was placed on *in vitro* three-dimensional models of epithelial cancers. We investigated questions such as the effect of matrix stiffening, activation of stromal cells, and identified potential advances in mechano-based therapies. Results: Meta-analysis revealed that 64% of studies report cancer invasion promotion as stiffness increases, while 36% report the opposite. Experimental approaches and data interpretations were varied, each affecting the invasion of cancer differently. Examples are the experimental timeframes used (24 h to 21 days), the type of polymer used (24 types), and choice of cell line (33 cell lines). The stiffness of the 3D matrices varied from 0.5 to 300 kPa and 19% of these matrices’ stiffness were outside commonly accepted physiological range. 100% of the studies outside biological stiffness range (above 20 kPa) report that stiffness does not promote cancer invasion. Conclusions: Taking this analysis into account, we inform on the type of experimental approaches that could be the most relevant and provide what would be a standardized protocol and reporting strategy.

## 1 Introduction

Cancer starts with mutations in one cell or a small group of cells. These mutations induce changes such as sustained proliferation, apoptosis resistance, and evasion of the host’s immune system.^37^ The cells escape the tissue’s homeostatic controls, hence becoming an independent, organ-like entity called tumor. The tumor consequently invades the surrounding tissue, breaching the vasculature and metastasising.^1^

The previously overlooked role and impact of stiffness and mechanical signaling in tumorigenesis is now starting to be recognized.^2,3^ There are key questions around the relationship between stiffness of tissue and cancer progression. These include how stiffness and invasion are correlated; the effect of stiffness on cancer cells; stromal cell contribution to the stiffness of the microenvironment; our ability to decouple stiffness from other physical parameters (such as plasticity and confinement) and, if so, how they individually affect cancer invasion; targeting stiffness as a potential therapy; and the use of tumor stiffness as a diagnostic tool.

Although previously published reviews of this topic exist,^3−8^ we aim to complement them by providing a systematic review of all literature published between 2004 and June 2020 on cancer invasion and stiffness. A unique meta-analysis using data extracted from our cohort of studies had been performed. In section 4, an exhaustive analysis of the *in vitro* methods used within the works reviewed is presented. Section 5 provides a critical analysis on the current consensus to questions such as correlation between stiffness and invasion (section 5.1), the role of stromal cells on matrix stiffening (section 5.2), the effect of other physical parameters (section 5.3) and anti-stiffness drug therapies (section 5.4).

## 2 Background

### 2.1 Defining Stiffness

Stiffness relates to how deformable a material is under a certain applied force.^9,10^ The elastic modulus, or Young’s Modulus (*E*), is used as a measure of this property. The associated unit is Pa (or N/m^2^) as it is derived from stress over strain. Stress and stiffness therefore have the same units since stress is a force per unit area (N/m^2^ or Pa) and strain a measure of normalized deformation (unitless). Although the Young’s modulus is the primary measure of elasticity in biology, the shear (*G*), storage (*G*′), and loss (*G*′’) moduli are also often evaluated.^10^ Approximative conversion between the Young’s modulus and the shear modulus is *E* ≈ 3*G*. The storage modulus can be approximated to the shear modulus at low frequencies, hence, *E* ≈ 3*G* ≈ 3*G*′. The loss modulus relates to the viscous (not elastic) properties of a material.

In tissue, stiffness is mainly dictated by the extracellular matrix (ECM). Increased ECM fiber deposition and increased cross-linking of these fibers correlate to stiffer tissues.^3^ Matrix density is regulated by fibroblast cells that, depending on microenvironmental cues, will either depose or degrade collagen fibers or remodel the existing ECM. The cross-linking degree is governed by enzymes such as lysyl oxidases (LOX) that catalyze covalent bonds between extracellular matrix proteins.^11^ Nonenzymatic collagen cross-linking such as glycation also stiffen the matrix.^12^ Other mechanisms of tissue stiffening are ECM fiber alignment, interstitial fluid pressure, strain stiffening due to forces applied by cells and cell jamming.^8^ Tissue stiffness range from 500 Pa for brain tissue^13^ to 20 GPa for cortical bone.^14^ Stromal tissues sit midrange, at 1–10 kPa.^3^

### 2.2 Measuring Living Tissues Stiffness

Biological tissues can be mechanically characterized on the macro, micro, or nano scale, *in vivo*, *ex vivo*, and *in vitro*. The most prevalent *in vivo* methods are shear wave elastography (SWE)^15,16^ and magnetic resonance elastography (MRE).^17,18^ As *ex vivo* measurements are easier to perform, a wider range of technologies are available. Commonly used techniques are atomic force microscopy (AFM),^19−23^ microindentation,^24,25^ and shear rheometry.^26^ It is, however, important to note that bulk stiffness, measured at large length scale (over millimeters) by SWE or MRE, is usually higher than local stiffness, measured by AFM or shear rheology, due to the heterogeneity of tissue components.^27^ For single cell level mechanical measurements, options are particle tracking micro rheometry,^28,29^ optical trap-based microrheology,^30,31^ micropipette aspiration,^32,33^ and AFM. Observation of fibers can indirectly serve as a stiffness measure, as increased deposition and alignment positively correlates to increased stiffness within the tissue. Second harmonic generation^34^ and scanning electron microscopy (SEM)^35^ are both tools that can be used to this effect. Guimarães *et al.* summaries all of the above methods in their review.^10^

### 2.3 How Do Cells Sense Stiffness?

Cells sense stiffness through mechanoreceptors. Mechanoreceptors are transmembrane proteins that transduce a mechanical cue outside the cell into a biochemical signaling. The main mechanoreceptors involved in ECM transduction are the integrin family.^36,37^ Integrins are linked to the cell’s cytoskeleton on the inside and bind to the ECM through focal adhesions on the outside. Activation of integrins by a mechanical cue in turn activates the Rho GTPase family that regulates cytoskeletal dynamics and intracellular contractile forces.^38^ Activation of integrins also signals to recruit talin, vinculin, and focal adhesion kinase (FAK), all of which participate in the formation of focal adhesions.^39^ Active integrins stimulate the production of transforming growth factor-β (TGFβ). TGFβ triggers the secretion of matrix proteins and matrix-modifying enzymes such as LOXs and matrix metalloproteinases (MMPs), which respectively cross-link and break down the matrix.^40^

General stiffness-dependent cell behavior has largely been demonstrated. Gene expression studies of mesenchymal stem cells plated onto matrices of different compliances have shown that matrix stiffness can drive cellular differentiation down alternative lineages.^41^ Endothelial cells form branched, capillary-like networks when cultured in a soft matrix but form larger tube-like structures with lumens in more rigid matrices.^42^ These examples highlight the vitality of integrating the mechanical microenvironment in any *in vitro* model.

### 2.4 Stiffness and Epithelial Tumor Invasion

**Figure 1:**
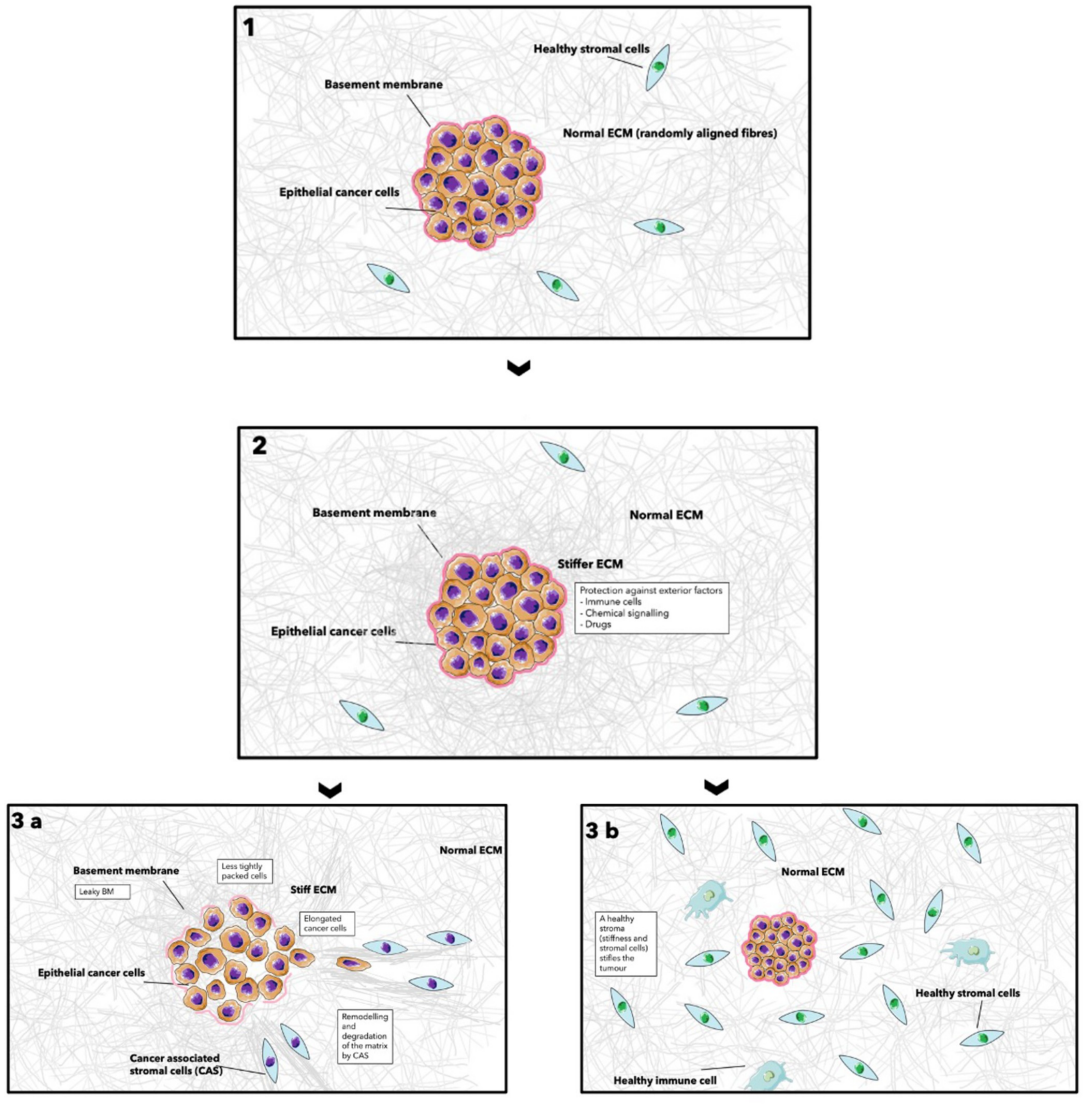
Evolution of onset epithelial cancer invasion. (1) The cancer cells are bound within a basement membrane. The healthy stroma that surrounds the tumor is composed of randomly aligned collagen fibers and healthy stromal cells such as fibroblasts, healthy epithelial cells, adipocytes and other organ specific cells. (2). The cancer cells signal to the stromal cells to stiffens the ECM at the tumor–stroma interface. This protects the tumor against exterior factors such as immune cells, chemical signaling and drugs. A gradient of stiffness appears, with the near ECM stiffer than the far ECM. The cancer can next either evolve into (3a) or (3b). This depends on various factors that are not yet fully understood. (3a) The cancer cells loosen their cell–cell adhesion and breach the basement membrane. Cancer-associated stromal cells aid by remodelling the ECM to create track for the cancer cells to invade along. The matrix is also locally degraded to facilitate cell migration. Cells change morphology; they elongate and become more flexible. The tumor as a whole is enlarge and loses its circularity. Cancer cells then migrate to the vasculature and metastasise. (3b) The near ECM is broken down to reduce stiffness (by directly reducing cross-linking and degrading fibers or by healthy stromal cells’ actions). The healthy environment stifles the tumor. Immune cells can now access the tumor to further suppress the tumor. Schematics were created using Servier Medical Art according to a Creative Commons Attribution 3.0 Unported License guidelines 3.0 (https://creativecommons.org/licenses/by/3.0/).

The evolution of epithelial cancer invasion, and its associated physical cues is illustrated Figure 1. The tumor signals to its microenvironment by releasing matrix modifying enzymes or by activating stromal cells. The tumor–stroma interface stiffens, which protects the cancer against exterior factors such as immune cells, chemical signaling, and drugs. The cancer cells loosen their cell–cell adhesion and breach the basement membrane. Cancer-associated stromal cells remodel the ECM to create tracks for the cancer cells to invade along. The matrix is also locally degraded to further facilitate cell migration.

Cells elongate and become more flexible. The tumor enlarges and loses its circularity. Cancer cells migrate to the vasculature and start metastasising. It has, however, been shown that reintroducing a healthy microenvironment, by targeting the matrix stiffness or by reprograming or reinstating healthy stromal cells, suppresses the tumor. Immune cells then access the tumor to further repress it.

**Figure 2:**
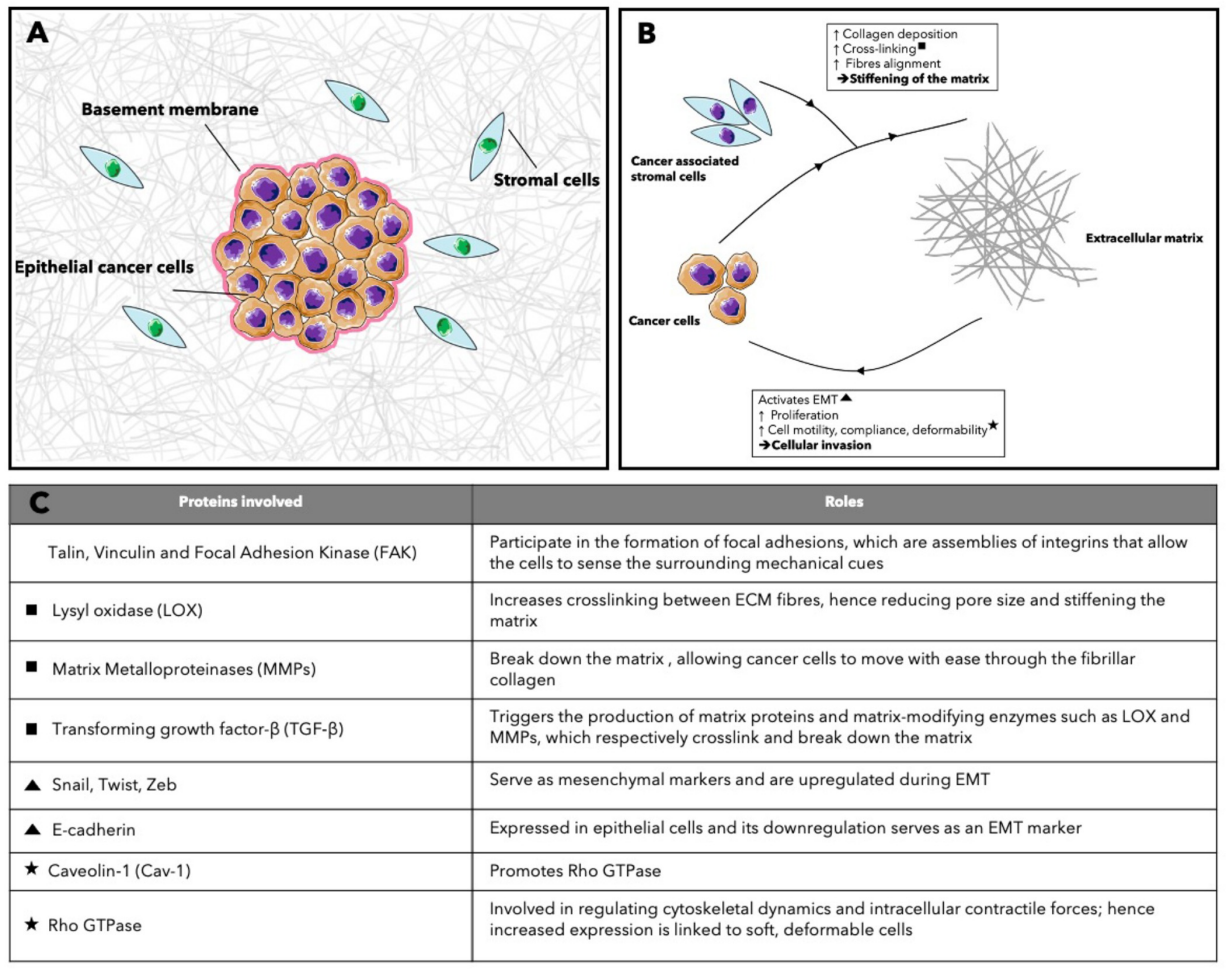
Stiffness and tumor invasion. (A) Schematic of an epithelial tumor, contained by a basement membrane and surrounded by a stroma. (B) Schematic of the cross talk between cancer cells and the surrounding stroma. Both cancer cells and stromal cells stiffen the matrix by increasing collagen deposition, cross-linking, remodelling, and alignment leading to matrix stiffening. The stiffened ECM in turn enhances cancer invasion by activating EMT, hence promoting proliferation and cell motility, compliance, and deformability. Schematics A and B were created using Servier Medical Art according to a Creative Commons Attribution 3.0 Unported License guidelines 3.0 (https://creativecommons.org/licenses/by/3.0/). (C) Table of proteins involved in this process. They can be related back to the schematic *via* the letterings in column two.

Now looking into more details of the biophysical and chemical cross-talk between cancer cells and the tumor microenvironment, the following is observed (Figure 2): The matrix initially stiffens due to the activation of TGFβ in newly mutated cancer cells. TGFβ in turns activates LOX enzymes, which increase cross-linking. TGFβ also affects the intracellular signaling, by modifying integrins^11,43^ and by upregulating the mesenchymal marker Snail, which downregulates E-cadherin,^44^ leading to the induction of epithelial-to-mesenchymal transition (EMT). EMT enables the cells to adopt mesenchymal behavior such as increased motility and therefore aids invasiveness.^45^ Stromal cells have an important role in remodelling the tumor microenvironment. Cancer-associated fibroblasts (CAFs) in particular participate in the desmoplastic reaction, which is an intense fibrotic response in tumors. CAFs lead the way of cancer invasion, by creating tracks of rearranged matrix for the cancer cells to follow.^46^ Perpendicularly arranged collagen fibers are linked to increased invasion, as they allow the cells to migrate more efficiently along them.^47^ Paradoxically, it appears that cancer cells soften and become more deformable, due to the overexpression of Rho GTPase.^38^ Deformability increases invasiveness as it allows the cells to move in the matrix with ease.

## 3 Systematic Literature Review Methodology

The aim of this review is to provide an overview of current research on the role of the physical microenvironment in cancer invasion. To gather information objectively, a systemic search of the available literature was performed. Particular focus was placed on *in vitro* three-dimensional (3D) models of epithelial cancers.

**Figure 3:**
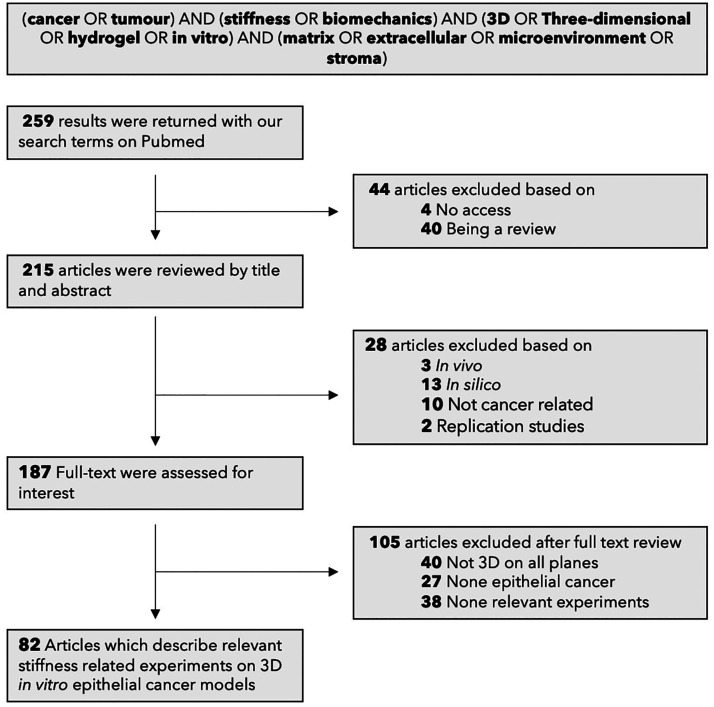
Systematic review flow diagram. Flowchart describing the systematic search strategy, including the identification, screening, and inclusion of relevant studies.

After refining specific search terms, the search was executed on the database Pubmed, which provided a highly relevant search output, with 259 results. The search terms used can be found in Figure 3. The search restricted dates to 2004–2020 and language to English only. The first exclusion pass used the following exclusion criteria: review papers, abstract only, and inability to retrieve a full copy of the paper. This reduced the search to 215 papers. After this first pass, remaining papers were reviewed for scientific quality and relevance. To later be able to directly compare the extracted information, the search was limited to *in vitro* 3D models of epithelial cancers. The following were therefore excluded: *in silico* and *in vivo* experiments, two-dimensional (2D) experiments only, nonepithelial cancers, and replication studies if the initial paper was also part of our cohort of studies. The process is illustrated in the PRISMA flow diagram Figure 3. The diversity in research focus and experimental procedures within these papers reduced the breadth of possible meta-analysis of the data. The review therefore provides both quantitative and qualitative assessments of current advancement in the field.

## 4 Methods Used for *In Vitro* Modeling Stiffness in Cancer Invasion

### 4.1 Three Dimensional over Two-Dimensional Models

**Figure 4:**
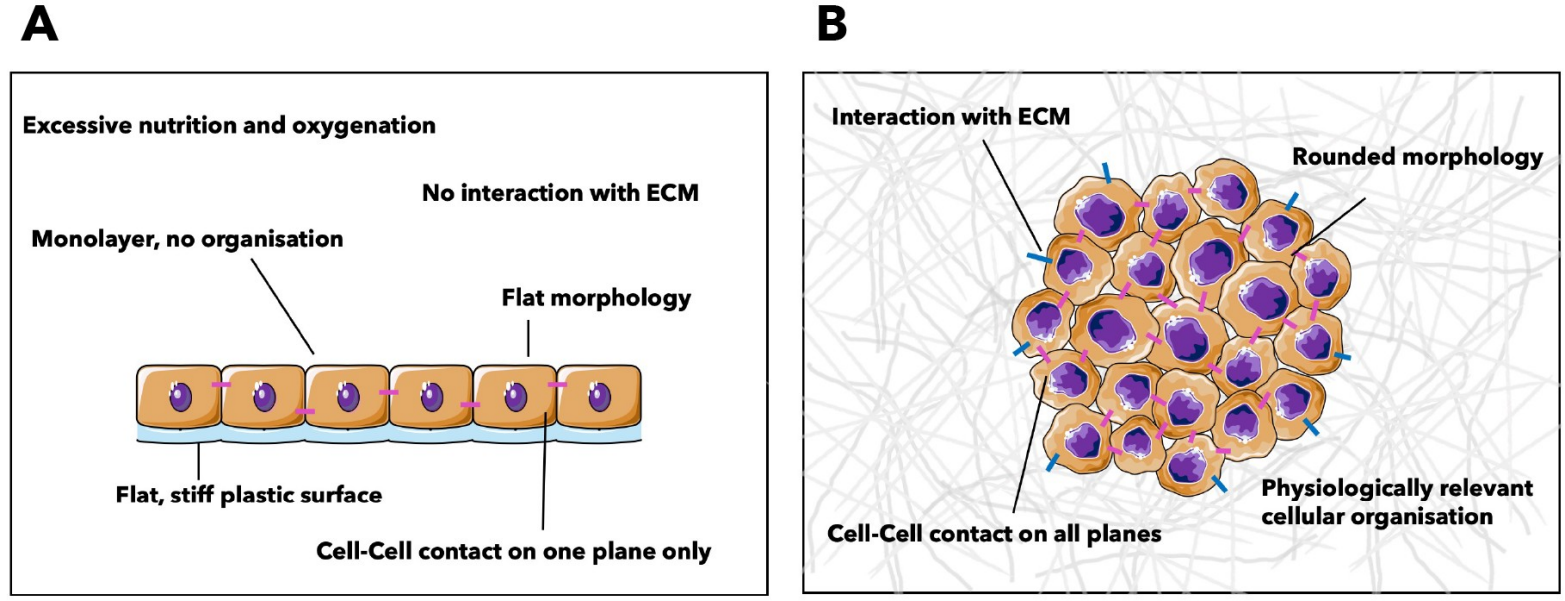
Two-dimensional and three-dimensional cell culture systems. Schematics of (A) a 2D culture system and (B) a 3D culture system. Difference in cellular organization and morphology are highlighted. Schematics were created using Servier Medical Art according to a Creative Commons Attribution 3.0 Unported License guidelines 3.0 (https://creativecommons.org/licenses/by/3.0/).

The first notable discordance in experimental protocols is whether to approach the problem with a two-dimensional (2D) or three-dimensional (3D) model. Traditionally, the vast majority of *in vitro* studies are performed in 2D. The ease and reproducibility of 2D culture has made working in this modality the previous standard for researchers. When studying the effects of the physical environment, the community conventionally uses 2D culture systems with substrates of varying stiffness.^48−53^ There are, however, inherent differences between 2D cultured cells and those in native tissue. The dissimilarities are notably reflected in morphology, migration speed, and cytoskeletal organization, as illustrated in Figure 4. Cells show flat morphology and organize in a monolayer when seeded on standard or hydrogel-coated Petri dishes. In contrast, cells embedded within 3D gels are characterized by a round shape and cluster organization.^22^ The geometry of adhesion within an ECM provides important cues that, for example, orientates polarity in epithelial cells.^54^ Breast carcinoma MDA-MB-231 cells and fibrosarcoma HT1080 cells have slower migration speeds in 3D than in 2D.^55^

Drug efficacy varies drastically when studied in 3D as opposed to 2D.^56,57^ Breast carcinoma cells become Doxorubicin resistant when transferred from a 3D model to a 2D culture.^57^ It has also been reported that 60% of compounds deemed efficient in 2D lack efficacy in humans during clinical trials.^58^ These examples highlight the necessity of cell–cell contact and adhesions to the matrix in a three-dimensional space for the cells to behave as they would in their physiological context. Therefore, employing 3D models is the most relevant and logical way of studying the effect of the physical microenvironment on cells.

### 4.2 Experimental Setup for Three-Dimensional Models

Synthetic polymers such as poly(ethylene glycol) (PEG) and polyacrylamide, though popular in tissue engineering, were only used in 14% of the papers reviewed. Physiologically relevant natural polymers such as collagen type I and Matrigel, a hydrogel derived from mouse tumor cells mainly consisting of collagen IV and unknown concentrations of other ECM components, were favored at 83% (see supplementary table S1). Contrary to the synthetic options, these natural polymers allow cells to sense the stiffness *via* integrin bindings, as well as remodel the fibers *via* LOX and MMPs. Common cell-surface receptors, including for VEGF, are expressed more when cells are attached to native ECM.^59^ While a natural environment should be preferred whenever possible, in some cases, it may be useful to effectively control parameters such as matrix density, pore size, and dynamic stiffening. Novel functionalized synthetic polymers allow such control while mimicking degradation and attachment, by incorporating a matrix metalloproteinase-degradable peptide cross-linker.^60,61^ Others favor synthetic–natural composite hydrogels.^62,63^ Note that all the polymers above will form matrices of fiber-based 3D meshes.

Aiming to increase the biomimicry of their models, certain groups replicate the basement membrane of epithelial cancers (carcinomas).^64,65^ The basement membrane is a barrier that compartmentalizes the tissue. Once breached by cancer cells, it becomes leaky and slowly vanishes. The membrane’s main component is collagen IV, making Matrigel favorable for its *in vitro* modeling. Most models combine the reconstituted basement membrane (rBM) with either collagen or alginate.

An important element of consideration is the control over the stiffness of the matrix. A simple way to stiffen a matrix is by varying the concentration of polymer. The literature shows that the stiffness of collagen I is the following: at a concentration of 1.5 mg/mL it is in the range 50–200 Pa; for a concentration of 4 mg/mL it is around 1000 Pa. A concentration of 4 mg/mL of Matrigel yields around 200 Pa.^66^ Another method used to increase collagen density, and therefore stiffness, is the use of plastic compression.^67,68^ The compression of a collagen hydrogel removes excess liquid, bringing the water content from 99% to 70%, therefore increasing the collagen percentage from <1% to 30%. This results in the creation of a more *in vivo* like environment.

Altering the stiffness by increasing polymer concentration modifies the pore size and the overall ECM structure, which can be unwanted. Increasing the cross-linking between fibers is an efficient way of stiffening the hydrogel while keeping the matrix density constant. Nonenzymatic glycation is often employed to this effect. Other stiffening technics include strain stiffening, where applying strain on collagen I can stiffen it 4-fold,^69^ and using synthetic polymers such as polyacrylamide. To integrate a temporal component to stiffness and invasion studies, cells can either be moved from low to high stiffness gels manually or integrated in a dynamically stiffening matrix. Staneva *et al.* showed that sugar threose allowed a temporal stiffening of a collagen I matrix over 24 or 48 h treatments.^70^ It allowed us to study whether the matrix stiffens pre or post onset invasion. Joyce *et al.* integrated calcium-loaded liposomes to drive gradual cross-linking of an alginate gel following exposure to near-infrared (NIR) light, allowing the matrix stiffness to change from 0.2 to 2 kPa.^57^ All the stiffness mentioned in the papers refer to the bulk matrix stiffness and not individual fiber stiffness. They were measured by methods such as rheology (at 44%) or AFM (31%). Further characterization of the matrix (*i.e.*, morphology, pore size porosity, fiber diameter) were not widely performed.

### 4.3 Choosing Cancer Cell Lines and Stromal Cells

Breast cancer represents 62% of all epithelial cancers reviewed in our cohort of studies. It is followed by colon and pancreas cancers (10% and 8% respectively), as represented in Figure 4. Each organ has unique cell behaviors, different stromal cells, different chemical cues, and different stiffnesses.

A common experimental strategy is to study two cell lines of varying invasiveness. In breast cancer, metastatic cancer cells are usually observed against nonmalignant mammary epithelial cells such as MCF10A or less invasive MCF-7 cells. Experiments using either MCF10A or MCF-7 report no response to stiffness increase, CAF activation, or other tumorigenic factors.^73^ Stromal cells are also often studied for their involvement in cancer invasion. Healthy and cancer-associated fibroblasts, as well as macrophages, T-cells and organ-specific cells such as pancreatic stellate cells, were the main stromal cells found within the scope of this review. It is important to note that although cell lines are mainly used for ease, they form spheroids that lack physiological genetic heterogeneity, which is known to contributes to cancer invasion.

Cancer cells are integrated in the 3D matrix as single cells mixed into the gel prepolymerization, which will form a spheroid within a day or two, or as preassembled spheroids. Stromal cells are studied alone, or are mixed with cancer cells, or are part of a more complex compartmentalization.

## 5 Current Prevalent Questions in Cancer Invasion Related to Stiffness

### 5.1 Effect of Stiffness on Cancer Invasion: Outcome Varies According to Stiffness Ranges, Temporality, Matrix, and Invasion Measures

**Figure 5:**
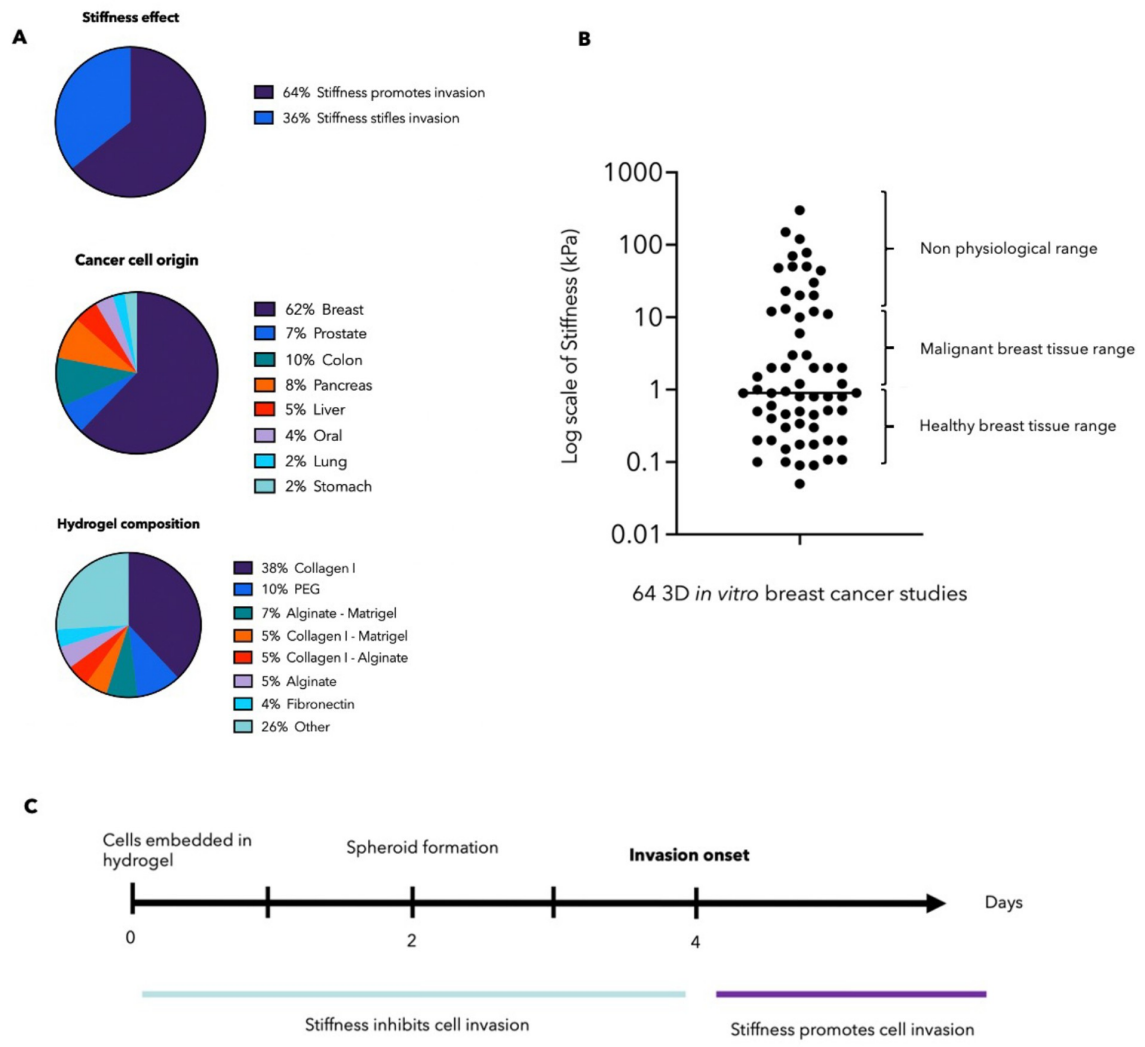
Analysis of the literature on cancer invasion related to stiffness. (A) The majority (64%) of studies observe that increased stiffness promotes invasion. However, a substantial amount of research (36%) reports the opposite. These results are all very dependent on the experimental procedure. The heterogeneity in strategies is reflected in the broad range of cancer type studies and hydrogel compositions. (B) More discrepancy is observed in the stiffness ranges studied. In over 64 3D *in vitro* breast cancer models, the matrix stiffness ranges from 50 Pa to 300 kPa with an average at 1 kPa (note the log scale). Healthy and malignant breast tissue, measured *ex vivo*, range from 0.3–1.83 kPa and 2–5 kPa, respectively.^14,21,63,64^ These values were all measured by either AFM or rheometer since our cohort of studies also used these instruments to measure the local stiffness. (C) Temporality also affects the outcome. Four days post seeding cells in the 3D matrix, cellular invasion is initiated. Observations before these 4 days will therefore majoritarily not report that stiffness promotes invasion.

The majority (64%) of studies observe that increased stiffness promotes invasion (Figure 5). General observations include a change in cell morphology, where cells elongate, and an increased proliferation rate within the tumor. The tumor loses its circularity, elongating as single cells detach and start migrating. The cells within the tumor are not as tightly packed, as they break their cell–cell junctions. The matrix is rearranged at the border of the tumor, fibers become parallel to the tumor, forming tracks for cells to migrate. In rBM models, the cancer cells need 3 days to diminish the basement membrane before invading. The above observations are more prominent in invasive cell lines, as opposed to their less invasive counterparts. However, a substantial amount of research (36%) reports that stiffness limits proliferation and invasion. These results are all very dependent on the experimental procedure adopted. As shown in Figure 5, the heterogeneity in strategies is reflected in the broad range of cancer type, matrix composition, and the stiffness range.

#### Stiffness Ranges

While Stowers *et al.* report that invasion is enhanced between a “soft” and “stiff” matrix, Cavo *et al.* observed the opposite.^22,72^ Upon further investigation, it is found that the former considers a soft gel to be 0.1 kPa and a stiff gel to be 1.2 kPa, while the latter describes 150 kPa to be soft and 300 kPa to be stiff. This major discrepancy and lack of clarity is commonly found in our systematic search of literature. Confusion also lies within the modulus reported. Stiffnesses are rarely reported with their type of modulus, which is concerning as elastic and shear/storage moduli differ by a factor of 3. Meta-analyses of the stiffness studies were therefore performed herein. Care was given to report all stiffness as the Young’s modulus, converting modulus when necessary. All models used for this analysis were breast cancer models as it represents the main cancer type within our cohort.

An initial analysis performed on 63 3D *in vitro* breast cancer models revealed that matrices were engineered to be anywhere between 50 Pa and 300 kPa, with a mean at 1 kPa. This is illustrated Figure 5. To put these stiffness ranges into perspective, we gathered data on healthy and malignant breast tissue stiffness, measured *ex vivo.* These papers were selected on the basis of their stiffness measurement technics, to be able to draw comparisons with the *in vitro* values. As in our cohort of studies, stiffness was measured by either AFM or rheometer. We concluded that healthy and malignant breast tissue range from 0.3 to 1.83 kPa and 2 to 20 kPa, respectively.^20,27,73,74^ This data can be found in supplementary table S2. It is therefore exposed that 19% of stiffness studies are out of physiological ranges, which we postulate to affect the invasion pattern of cancer.

A second analysis of 41 papers, for which both stiffness and outcome could clearly be extracted and related, revealed that 100% of studies using nonphysiological matrices (>20 kPa) observed that invasion is inhibited (Figure 5). In the *in vivo* stiffness range (0–20 kPa), invasion is mostly promoted with increased stiffness. Interestingly, this briefly changes 3 and 10 kPa. A hypothesis is that the limitation lies in the pore size of matrices of high stiffnesses. Indeed, increasing the stiffness by increasing the matrix density also reduces the pore size. Pore sizes smaller than ∼5 μm become a significant limiting factor as this is the size of an epithelial cell’s nucleus. Section 5.3 explores in more depth the relationship between invasion and confinement. Matrices of over 10 kPa are mainly stiffened with methods that does not increase the matrix density, as briefly described in section 5.3. The pore size is no longer limiting, which we postulate explains why stiffness promotes invasion again.

#### Temporal Role

Temporality plays a critical role in the outcome observed. Staneva *et al.* report that if the stroma is stiff before onset invasion, infiltration is dramatically inhibited.^70^ However, when the matrix is stiffened after the onset of invasion, it increases 2-fold compared to the control value. These findings are reflected within the collective effort to implement temporal stiffening of the matrix.^57,75^ Cellular invasion is initiated 4 days after seeding cells in the 3D matrix. Observations before these 4 days will therefore likely not report that stiffness promotes invasion, and these results would not reflect on the actual infiltration of the cancer cells. It was also observed that the higher the stiffness, the more delayed the invasion is.^76^Figure 5 summarizes the effect of temporality on invasion.

#### Matrix

The choice of hydrogel also has an important influence on the outcome. Experiments using synthetic polymers understandibly differ from natural polymer as they do not allow integrin mediated mechanotransduction, matrix remodelling and degradation. Different invasion patterns were also observed among natural hydrogels. Collagen, being the ECM’s main component, unsurprisingly yields the most biomimetic results. Carey *et al.* compared collagen I to Matrigel and showed that as collagen I content was increased and Matrigel content decreased, organoids became increasingly invasive, losing their rounded morphology and becoming stellate and protrusive.^77^ Collagen I uniquely initated EMT and upregulated MT1-MMP expression, whereas cells in Matrigel significantly downregulated MT1-MMP. These results are consistent with the fact that MT1-MMP specifically breaks down collagen matrix.“Matrigel only” (rBM), “col:rBM”, and “collagen only” matrices of same stiffness (approximately 800 Pa) show significantly different cluster circularity.^65^ Cell clusters in “collagen only” gels were more than half as circular (circularity of 0.3 as opposed to 1), which implies that they are more invasive.

#### Invasion Measures

A lack of standardization of invasion evaluation strategies was noticeable between research articles. Cluster area, cluster diameter, number of invading cells, circularity, motility, proliferation rate, polarization, expression of E-Cadherin, and fraction of dispersed single cells, were all used as measures of invasion. Normalization to a control is necessary, as invasion of the cells will eventually arise independently of stiffness. It was found that ECM stiffness does not necessarily induce tumor cell invasion but accelerates organoid dissociation.^78^ Spheroids incorporated into a soft matrix were reported to increase significantly in size over 3 days, but individual MDA-MB-231 cells did not dissociate from the spheroid body.^71^ Therefore, while cluster size and proliferation generally inform on the aggressiveness, invading cells count, circularity, and motility are a better direct reporter of invasion.

### 5.2 Role of Stromal Cells in Matrix Stiffening: A Two-Way Process

Cancer cells do not drive matrix remodelling alone. They recruit nonmalignant stromal cells from their microenvironment and induce a change in their phenotype making them cancer-associated stromal cells (CASCs). In addition to a cascade of biochemical signaling between cancer cells and CASCs that influence cancer cell invasion, CASCs promote invasion by remodelling the matrix or by mechanical coupling to the cancer cells. Healthy stromal cells have the opposite effect on the tumor; normal immune cells suppress cancer cells and normal fibroblasts reduce the ECM stiffness and diminish cancer invasion.^71^ The recruited stromal cells range in type and include vascular endothelial cells, adipocytes, fibroblasts, organ specific stromal cells and mesenchymal stem cells (see Bussard *et al.* for a review on tumor-associated stromal cells^79^).

Cancer associated fibroblasts (CAFs) are of particular interest as they are responsible for collagen maintenance, deposition, and reabsorption. Common CAF activation markers are α-smooth muscle actin (αSMA), paladin, and caveolin-1.^71,81^ At early time points, healthy fibroblasts in a soft matrix (100 Pa) upregulate CAFs markers. Stiffer matrix (800 Pa) prevents them from doing so, and physically entraps the fibroblasts that have not yet acquired cancer-associated phenotypes.^71,82^ At later time points, stiffness activates CAFs.^81^ CAFs effect on matrix remodelling is significantly increased when in the presence of tumor cells.^71^ TGF-β treatment of an *in vitro* 3D pancreatic cancer model activates elongation, cell spreading, lamellipodia formation of CAFs and spheroid invasion but has no effect on healthy fibroblasts.^83^ TGF-β is a growth factor commonly released by cancer cells and is showed to have effect on production of matrix-modifying enzymes such as LOX and MMPs.

The altered tumor ECM also affect immune cells. The stiff, collagen-rich near tumor–stroma border physically impedes T cell infiltration. The high-density matrix also reduces T cells proliferation and downregulates cytotoxic activity markers.^84^ Conditioned media from macrophages promotes tumor cell growth in high stiffness (1 kPa). No difference is observed in low stiffness (100 Pa).^85^ This shows that the macrophages become tumor associated in pathological stiffness ranges. Senescent mesenchymal stem cells (MSCs), although less motile than presenescent MSCs, remodel the ECM driving breast cancer cells to a more-invasive phenotype.^86^ Pancreatic stellate cells (PSCs) cultured in a stiff matrix (10 kPa) expressed higher levels of αSMA and CTGF which are both PSCs activation marker.^87^

### 5.3 Elasticity, Plasticity, Matrix Density, Confinement, And Alignment

Biophysical cues such as elasticity, plasticity, viscoelasticity, matrix density, fibers diameter, alignment, and cell confinement all contribute to the mechanical microenvironment. Researchers have been trying to decouple each of these components to understand their individual impact on cancer migration. Plasticity, as opposed to elasticity, is a material’s ability to undergo nonreversible deformation. Plasticity tuning alone shows the same pattern as for stiffness: MDA-MB-23 cells in high plasticity are more migratory and have a more elongated morphology.^64^ Collagen I alignment, created by applying a uniaxial tension to the matrix or rotational alignment, directs migration, a process called contact guidance, enhancing the efficiency of cancer invasion and metastasis.^90,91^

The physical confinement of cells independently induces malignancy induction.^92,93^ Experiments with hydrogel-microchannels of tunable stiffness and confinement show that confinement induces EMT even in the cell clusters surrounded by a soft matrix, which otherwise protects against EMT in unconfined environments. However, when combining stiff matrix and confinement, a transition was observed, with cell migration in a more aggressive amoeboid mode.^94^

**Figure 6:**
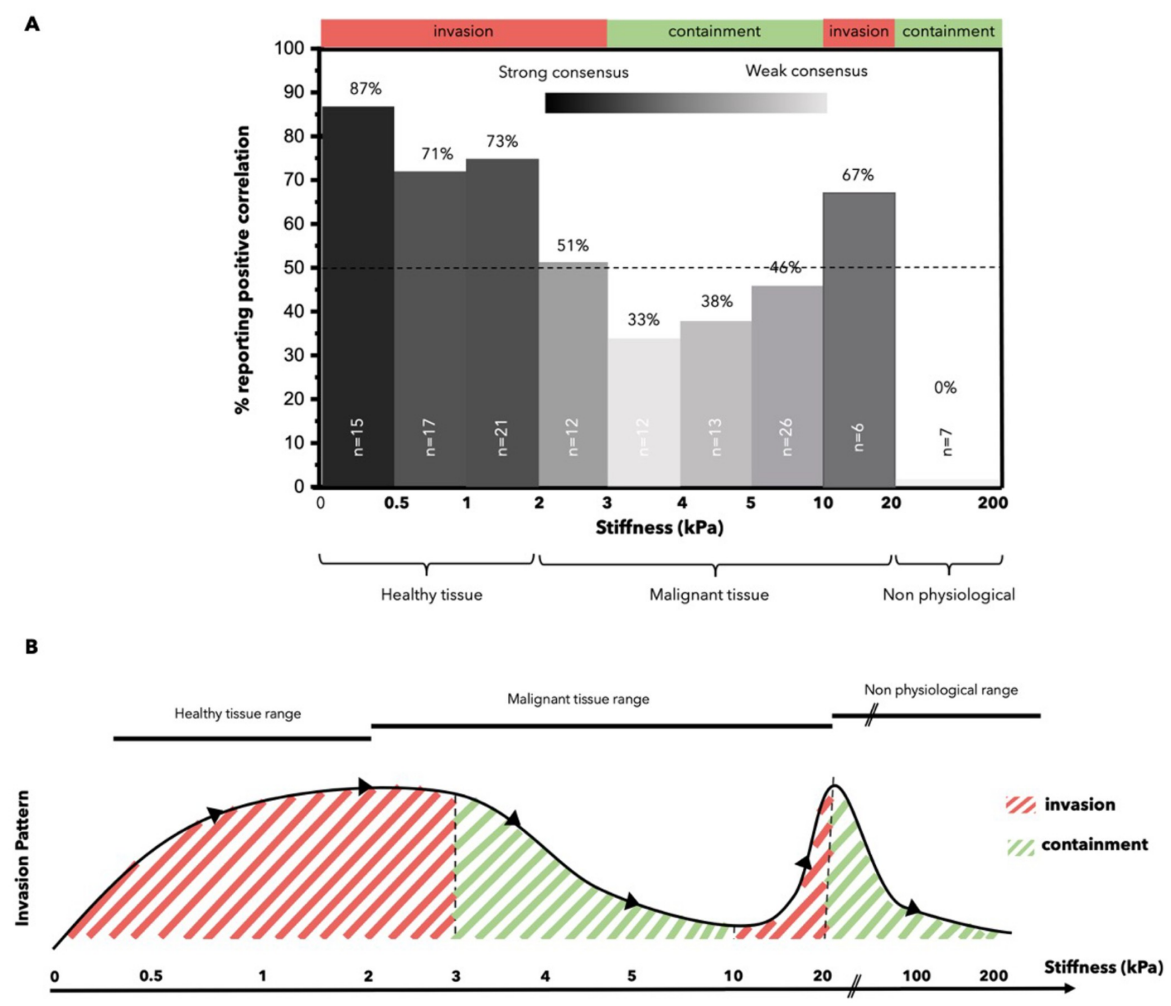
Invasion patterns vary across stiffness ranges. Outcome of an analysis of 41 papers, where stiffness and invasion pattern could be correlated. (A) Plot reporting the percentage of papers showing a positive correlation between increase of stiffness and increase of invasion. (B) Visual summary of whether each stiffness range promotes cancer invasion or not. The *y* axis represents a trend and is not numerical. 100% of studies using nonphysiological matrices (>20 kPa) observed that invasion is inhibited as stiffness increases. In the *in vivo* stiffness range (0 to 20 kPa), invasion is promoted as stiffness is increased. This briefly changes between 3 and 10 kPa. A hypothesis that the limitation lies in the pore size of the matrix. Indeed, increasing the stiffness by increasing the matrix density also reduces the pore size. Pore sizes smaller than ∼5 μm become a significant limiting factor as this is the size of the nucleus of epithelial cells.

There is, however, a limit to which confinement promotes invasion. Pradhan *et al.* hypothesized that above 5 kPa, the pore size is smaller than a cancer cell nucleus (3–5 μm in diameter), which implies that the cell can no longer migrate through the mesh.^62^ Cassereau *et al.* observed a threshold of 5 mg/mL of collagen, or 3 kPa, after which the increased rigidity did not promote invasion.^78^ Decoupling the effects of stiffness and fiber density *via* a tension bioreactor platform, they observed that when pore size was not limiting, stiffness could, independently, further enhance cell migration.^78^ This may explain the change in trend observed in Figure 6 where after 3 kPa, invasion is no longer promoted *in vitro* although *in vivo* values suggested that it should be. The high matrix density along with the limitations of *in vitro* models (limited duration of experiments, low degradability of some matrices, absence of stromal cells) does not allow degradation by MMPs and rearrangement of the matrix to allow cells to overcome pore size limitation.

### 5.4 Matrix Stiffening and Anticancer Drugs: Stopping Cancer Invasion by Targeting Matrix Stiffness

With the recognition of the role of the physical environment in cancer progression, mechano-based therapies that target increased tissue stiffness are emerging clinically. The therapies target the ECM itself by causing degradation (MMPs activation or bacterial collagenase) or inhibiting cross-linking (LOX inhibition). They can also target the integrins to limit cancer cell sensing or target the stromal cells. This is called stroma-reprogrammed combinatorial therapy (SRCT). Reprogramming stromal cells in a stiffened microenvironment leads to reduced “barrier effects” and increased tissue-infiltration of the chemotherapy drug.^95^ A high-throughput mechano-pharmacological screening platform has been developed for SRCT.^95^ Tranilast and Doxorubicin have been tested on a complex compartmentalized cancer-stroma model and reduced fibrosis and condensed tumor growth and invasion.^96^ MDA-MB-231 cells have a stiffness-dependent resistance to doxorubicin.^57^ The fibrous physical barrier around the tumor therefore is not the lone cause to resistance, as the stiffness induces a phenotypic change in cancer cells. Less invasive MCF7, however, does not exhibit such dependency. Targeting the barrier effect is not the only way of utilizing cancer stiffness. Anticancer drug delivery has been achieved by sending ultrasoft (0.1 kPa) cell-sized microparticles in the stroma. The microparticles are successfully integrated as part of the spheroid and can then locally deliver the drug.^98^

## 6 Conclusion

Although 64% of research articles within our systematic review report that increased stiffness can directly be linked to invasion promotion, the thirty-six other percent report opposite findings. The inconsistency in experimental approaches we observed within our cohort of studies contributes to explaining this. We perceived high variability in matrix polymer, duration of experiments, cell lines, and stiffness of the matrix, all of which is summarized in Table 1. Certain of these experimental choices limit the biomimicry of the cancer model and all affect the experimental outcome. We also observed that poor reporting of invasion measures, stiffness measurement instruments and modulus hinders the comparability of each experiment.

**Table 1:** Experimental Parameters and How They Vary within the 81 Studies Part of This Review’s Cohort^a^

experimental parameter	variability found within the studies
matrix polymer	24 different types 14% synthetic vs 86% natural
experiment duration	from >24 h to 21 days
cell-lines	33 different types
matrix stiffness	from 50 Pa to 300 kPa 19% of stiffness outside of physiological range
stiffness measurement methodology	44% rheology, 31% AFM

a This summarizes data found in Figure 5 and supplementary tables 1 and 4.

We propose a standardization of protocol and reporting strategy. On the basis of the research reviewed herein, a collagen matrix allows for the most biomimetic tumorigenesis of epithelial cancers. Cells may then sense their surroundings *via* integrins, and all relevant matrix modifying proteins can take effect.

This is of particular relevance when studying the effect of the physical microenvironment. We estimate that measurements should be taken day 7 or later, to ensure that the cells have had time to form a spheroid, and sense and react to their environment. A progressive stiffening of the matrix would further help. Models of healthy and malignant tissues should match their physiological stiffness ranges, which is approximatively 100 Pa to 2 kPa for the former and 1 to 20 kPa for the latter. This range fits stromal tissues, although it will slightly vary depending of the organ. Stiffness should be reported with its corresponding moduli. Numerical values to describe the stiffness should be favored instead of using relative terms such as “soft”, “compliant” or “stiff”. The community would benefit from a standardized invasion measurement system.

Overall, this review provides a summary of the current literature available on using 3D *in vitro* models for investigation the role of stiffness in cancer invasion. The systematic approach allows for a complete and unbiased read of all the literature. The main topics researchers are currenting looking at are how stiffness and invasion are correlated; stromal cell contribution to the stiffness of the microenvironment; decoupling stiffness from other physical parameters and targeting stiffness as a potential therapy.

## Supporting Information Available

Tables of matrices and frequency of use, stiffness of healthy and malignant *ex vivo* breast tissue, correlation between invasion promotion and stiffness, and cell lines used for 3D *in vitro* modelling of cancer invasion (PDF)
