# Supplementary material for: 3D In Vitro Models for Investigating the Role of Stiffness in Cancer Invasion
Source: ACS Biomater Sci Eng. 2021 Jun 3;9(7):3729–41. doi: 10.1021/acsbiomaterials.0c01530 (PMC10336749; doi:10.1021/acsbiomaterials.0c01530)
Supplement: Supplementary file 1 — ab0c01530_si_001.pdf [file ab0c01530_si_001.pdf]

SUPPORTING INFORMATION FOR PUBLICATION

3D IN VITRO MODELS FOR INVESTIGATING THE ROLE OF STIFFNESS IN CANCER INVASION

Auxtine Micalet[1,3], Emad Moeendarbary[1,2], Umber Cheema[3*]

[1]Department of Mechanical Engineering, University College London (UCL)

[2] Department of Biological Engineering, Massachusetts Institute of Technology (MIT)

[3]Division of Surgery and Interventional Sciences, UCL Centre for 3D Models of Health and Disease, University College London (UCL)

*Corresponding author:

Umber Cheema (u.cheema@ucl.ac.uk)

Mailing Addresses:

Auxtine Micalet and Emad Moeendarbary:

University College London, Mechanical Engineering

Roberts Engineering Building, University College London, Torrington Place,

London, UK WC1E 6BT

Umber Cheema:

University College London, Division of Surgery and Interventional Sciences

UCL Centre for 3D Models of Health and Disease

Charles Bell House,

London, UK W1W 7TS

5 PAGES, 4 TABLES

A

Matrix composition	Number of studies	Frequency
2PL	1	1%
Agarose	1	1%
Agarose - Matrigel	1	1%
Alginate	4	5%
Alginate - Matrigel (interpenetrating network)	6	7%
Col-Tgel	2	2%
Collagen - Alginate	4	5%
Collagen - Fibronectin	1	1%
Collagen - Gelatin	1	1%
Collagen - Matrigel	4	5%
Collagen - PEG	2	2%
Collagen I	31	38%
Denaturalized ECM	2	2%
Fibroblast Derived Matrix	2	2%
Fibronectin	1	1%
Gelatin	3	4%
Gelatin - HA	1	1%
Hyaluronic Acid - Matrigel	1	1%
Hyaluronic Acid	1	1%
Matrigel	1	1%
Polyacrylamide - PEG	1	1%
PEG	8	10%
PEG - Fibronectin	1	1%
Polyacrylamide	1	1%
	TOTAL STUDIES: 82	

B

Matrix type		Frequency
Natural		83%
Synthetic		14%
Natural co Synthetic		4%

S1 – A) Table listing all matrices polymers and their frequency of use. **B)** Table comparing frequency of use of natural vs synthetic vs natural co synthetic matrices.

Tissues	Values (E; kPa)	Values (G, kPa)	Instrument	References
Breast-Healthy	1.13 - 1.83		AFM (E)	Plodinec, M., Loparic, M., Monnier, C. A., Obermann, E. C., Zanetti-Dallenbach, R., Oertle, P., … Schoenenberger, C.-A. (2012). The nanomechanical signature of breast cancer. Nature Nanotechnology, 7(11), 757–765. https://doi.org/10.1038/nnano.2012.167
Breast-Healthy	0.3	0.1	Rheology (G)	Levental, K. R. et al. Matrix Crosslinking Forces Tumor Progression by Enhancing Integrin signaling. Cell 139, 891–906 (2009).
Breast-Healthy	0.4		AFM (E)	Acerbi, I., Cassereau, L., Dean, I., Shi, Q., Au, A., Park, C., … Weaver, V. M. (2015). Human Breast Cancer Invasion and Aggression Correlates with ECM Stiffening and Immune Cell Infiltration HHS Public Access. Integr Biol (Camb), 7(10), 1120–1134. https://doi.org/10.1039/c5ib00040h
Breast - Cancer	2.7	0.9	Rheology (G)	Levental, K. R. et al. Matrix Crosslinking Forces Tumor Progression by Enhancing Integrin signaling. Cell 139, 891–906 (2009).
Breast - Cancer	2-20		AFM (E)	Plodinec, M., Loparic, M., Monnier, C. A., Obermann, E. C., Zanetti-Dallenbach, R., Oertle, P., … Schoenenberger, C.-A. (2012). The nanomechanical signature of breast cancer. Nature Nanotechnology, 7(11), 757–765. https://doi.org/10.1038/nnano.2012.167
Breast - Cancer	2-15		AFM (E)	Zanetti-Dällenbach, R. et al. Length Scale Matters: Real-Time Elastography versus Nanomechanical Profiling by Atomic Force Microscopy for the Diagnosis of Breast Lesions. (2018) doi:10.1155/2018/3840597.
Breast - Cancer	>5		AFM (E)	Acerbi, I., Cassereau, L., Dean, I., Shi, Q., Au, A., Park, C., … Weaver, V. M. (2015). Human Breast Cancer Invasion and Aggression Correlates with ECM Stiffening and Immune Cell Infiltration HHS Public Access. Integr Biol (Camb), 7(10), 1120–1134. https://doi.org/10.1039/c5ib00040h

S2 Table listing stiffness of healthy and malignant *ex vivo* breast tissue. Studies are independent from the systematic search. This data was gathered to put into context the stiffness ranges used for 3D *in vitro* cancer models. All stiffness measurements were made using either AFM or rheology as to be able to relate these values to the *in vitro* model values.

Stiffness (kPa)	Is invasion positively correlated with stiffness?	Yes %	Total number of data points
0 - 0.5	**YES**	87	15
0.5 -1	**YES**	71	17
1 - 1.5	**YES**	80	15
1.5 - 2	**YES**	67	6
2 - 3	**YES**	51	12
3 - 4	**NO**	33	12
4 - 5	**NO**	38	13
5 - 6	**NO**	43	7
6 - 7	**/**	50	6
7 - 9	**NO**	40	9
9 -10	**/**	50	4
10 - 20	**YES**	67	6
20 -300	**NO**	0	7

S3 Correlation between invasion promotion and stiffness. For each stiffness range in column one, we have recorded whether the majority of studies found a positive correlation between stiffness and invasion or not.

Organ	Cell lines	Total number of time used in studies
**Liver**	Huh7	3
	HepG2	1
	Hep3B	1
**Breast**	MDA-MB-231	26
	MCF7	8
	4T1	3
	MCF10A	13
	SKBR3	2
	MDA-MB-361	1
	MDA-MB-453	1
	Hs578T	1
	HCC1806	1
	SCg6	1
	T47D	1
**Prostate**	PC-3	3
	LNCaP	1
	DU145	1
	VCaP	1
	C4-2B	1
**Colon**	HCT-116	4
	CT26	1
	SW620	1
**Pancreas**	CFPAC	2
	COLO-357	1
	MIA PaCa-2	1
	Suit2-007	1
	BxPC-3	1
	PANC-1	1
**Oral**	Tca 8113	3
	SAS	1
**Lung**	A549	2
	H1299	1
**Stomach**	AGS	2

S4 Table listing all the cell lines used for 3D *in vitro* modelling of cancer invasion
